# Editorial: Explanation in human-AI systems

**DOI:** 10.3389/frai.2022.1048568

**Published:** 2022-10-26

**Authors:** Anastasia Angelopoulou, Epaminondas Kapetanios, David Harris Smith, Volker Steuber, Bencie Woll, Frauke Zeller

**Affiliations:** ^1^College of Design, Creative and Digital Industries, School of Computer Science and Engineering, University of Westminster, London, United Kingdom; ^2^School of Physics, Engineering and Computer Science, Department Computer Science, University of Hertfordshire, Hatfield, United Kingdom; ^3^Department of Communication Studies and Multimedia, McMaster University, Hamilton, ON, Canada; ^4^Deafness Cognition and Language Research Centre, University College London, London, United Kingdom; ^5^School of Professional Communication, Toronto Metropolitan University, Toronto, ON, Canada

**Keywords:** artificial intelligence, machine learning, explainability and interpretability, human-computer interaction, natural language processing

Autonomous vehicles, social and industrial robots, image-based medical diagnosis, voice-based knowledge and control systems (e.g., Alexa, Siri), and recommendation systems are some application domains, where AI/ML-assisted digital artifacts already support daily routines and activities. Explainable Artificial Intelligence (XAI) and Interpretable Machine Learning (IML) are both terms coined as a response to the proliferation of automated systems penetrating societal, economic, industrial, and scientific environments by concerned users. For instance, what is a *good explanation* when an autonomous car crashes? What is a good explanation when Alexa is asked to explain *why inflation is so high in the UK* and the usual response is hilariously inappropriate? What is a good explanation when social media giants make recommendations for followers and for adverts to lay users?

These concerns, particularly in mission-critical or life-affecting systems, are fuelled by the increased complexity of such systems, which inevitably turn them into a “black box” when it comes to explaining and interpreting their decisions, outcomes, or behavior. Despite the fact that interesting algorithmic approaches and implementations, e.g., LIME, IBM Protodash, and SHAP (SHapley Additive exPlanations), have been taken to tackle this challenge to look through the “black box” and understand its behavior, these have been predominantly driven by ML developers, and are designed to improve the ML models rather than to provide some explanation or interpretation of the outcome for the consumer of the system.

The answer to the question “what is a good explanation for lay users” becomes more challenging within a broader, multi-disciplinary context, as the one of our call, from philosophy to sociology, economics, and computer sciences. In this context, XAI should entail another level of discussion that need to be addressed: our relationship as humans, to AI systems, in general. Nonetheless, the term “explain” derives from the Latin verb “explanare”, which means, literally, “to make level”. Thus, the discussions and scientific endeavors into XAI could entail the notion of ourselves “making level” with AI systems, which again brings us to the old question as to our relationship to and with AI systems. More specifically, bringing in our multifaceted cultural notions of AI and human beings, for instance, in terms of master and servant, or who is and will be dominating whom (e.g., Space Odyssey's HAL).

Arguably, the intertwined cultural perceptions and (often incorrect, albeit popular) ideas regarding AI and its potential ultimately also influence lay persons perceived needs for explanation when interacting with AI systems. For example, when interacting with a social robot, ideally, we should not have any perceived need for explanation—or only as much or little as we would have when interacting with any other social, human companion. Given that we are seeing a machine, however, brings up expectations and impressions formed by popular culture, and thus the need for explanation to, maybe, satisfy a need for safety, trust, etc.

Therefore, answering the research question “what is a good explanation” is far from obvious. Seeking answers to this research question has been the main incentive for the launch of this Research Topic. Even though specific answers to this research question cannot be given, general research directions and perspectives for seeking answers have emerged from the seven contributing articles.

In a nutshell, the key message has been that it is impossible to provide answers to this research question without consideration of at least the following aspects: (a) Philosophical foundations of what an explanation is (e.g., causality, interpretability and justification, structure of explanations); (b) Social attribution: how people explain behavior (e.g., intentionality and explanation, beliefs, desires, intentions); (c) Cognitive processes underpinning how people explain and evaluate explanations (e.g., cognitive bias, norms and morals, mutability of explanations, counterfactuals); (d) Social explanation, or how people communicate explanations (e.g., explanation as conversation and dialogue—whether in a spoken or signed language—or explanations using non-linguistic means).

More specifically, the following key insights should be acknowledged.

From the article “*3Es for AI: Economics, Explanation, Epistemology*” (Original Research Article), we learn that there is a differentiation between ordinary and scientific meanings of explanation; *while ordinary explanation replaces the unfamiliar by the familiar, scientific explanation replaces the familiar by the unfamiliar* (Kaul). Hence, a “good explanation” may not be necessarily “comprehensible”, if it is true. Furthermore, the *concept* of explanation, as of the example of explanations in economics, may mutate and inflect, indicating the changing ways in what counts as knowledge and the terms of access to it have been understood.

From the article “*Explainable Model Fusion for Customer Journey Mapping*” (Original Research Article), we understand that, in lay terms, economics and business are often conflated, hence, the answer may lie with *customer journey mapping as an approach to explaining and understanding behavior in a market-driven economy* (Okazaki and Inoue).

From the article “*Self-Explaining Social Robots: An Explainable Behavior Generation Architecture for Human-Robot Interaction*” (Original Research Article), we take the message that before even asking ourselves “*what is a good explanation?*”, we should design and create systems capable of explaining themselves first (Stange et al.).

From the article “*Prediction, Knowledge, and Explainability: Examining the Use of General Value Functions in Machine Knowledge*” (Perspective Article), we learn that, perhaps the answer to this question may lie with the “agent self-assessing its learning environment by carrying out model estimates and calculating the certainty in those estimates during decision-making” (Kearney et al.).

From the article “*Benchmarking Perturbation-Based Saliency Maps for Explaining Atari Agents*” (Original Research Article), we learn how difficult it is for agents to explain their actions when they rely on highly complex ML models such as Deep Reinforcement Learning and Saliency Maps (Huber et al.).

From the article “*GANterfactual—Counterfactual Explanations for Medical Non-experts Using Generative Adversarial Learning*” (Original Research Article) we may take the insight that despite the diversifying aspects in providing an answer to our research question, perhaps the creation of counterfactuals may the best approach to explain things to lay users (Mertes et al.).

From the article “*AI Technologies, Privacy, and Security*” (Hypothesis and Theory Article), we may take the message that issues surrounding privacy and data protection may limit the depth and breadth of explanation (Elliott and Soifer).

## Author contributions

All authors have been listed in alphabetical order. All authors listed have made a substantial, direct, and intellectual contribution to the work and approved it for publication.

## Conflict of interest

The authors declare that the research was conducted in the absence of any commercial or financial relationships that could be construed as a potential conflict of interest.

## Publisher's note

All claims expressed in this article are solely those of the authors and do not necessarily represent those of their affiliated organizations, or those of the publisher, the editors and the reviewers. Any product that may be evaluated in this article, or claim that may be made by its manufacturer, is not guaranteed or endorsed by the publisher.

